# Evidence on the social, economic, and environmental impact of interventions that facilitate bamboo industry development for sustainable livelihoods: a systematic map protocol

**DOI:** 10.1186/s13750-022-00286-8

**Published:** 2022-10-20

**Authors:** Lucy Binfield, Tamara L. Britton, Chunping Dai, John Innes

**Affiliations:** 1 Department of Forest Resources Management, Faculty of Forestry, University of British Columbia, V6T1Z4 Vancouver, British Columbia Canada; 2 Department of Anthropology, Centre for Environment and Sustainability, Faculty of Social Science, Western University, N6A 3K7 London, Ontario Canada; 3 Department of Wood Science, Faculty of Forestry, University of British Columbia, V6T1Z4 Vancouver, British Columbia Canada

**Keywords:** Program evaluation, Non-timber forest products, Social impact, Nature-based solutions, Theory of change.

## Abstract

**Background:**

Bamboo has been identified as a potential instrument for socioeconomic development due to its fast growth, perceived environmental benefits, promising material properties, myriad applications, and relative underdevelopment as a global industrial product. Many projects and interventions have been carried out that aim to utilize bamboo’s social and environmental potential in development. However, critical evaluations that demonstrate this effect using real-world evidence and outcomes are rare, and existing case studies have not been collated and analyzed in a systematic way. The proposed systematic map aims to summarize and evaluate evidence on the social, economic, and environmental impact of bamboo industry development initiatives on beneficiary communities and ecosystems, and to identify priority areas for future funding and research.

**Methods:**

In the proposed systematic map, we will collect and thematically categorize evidence on the social, environmental, and economic impact of bamboo development implementations, identifying themes, research gaps, and critical success factors. Literature discussing this type of intervention is published by researchers, organizations, and governments in academic journals, institutional reports, and program evaluations describing various socio-economic and environmental outcomes, impacts and metrics for success. Search sources for this systematic map therefore include bibliographic databases, institutional websites, web-based search engines, and expert consultation. Targeted search strings will be used to identify relevant texts in a two-step review process comprising an abstract and a full-text screening process. Sources describing interventions with a primary aim of bamboo industry development for social benefit that concluded between 1990 and 2021 will be sought. Metadata coded from these texts will be reviewed, categorized, and checked by two reviewers. Reviewers will be checked for consistency on batches of 30 articles using the Kappa interrater reliability test with a goal of a Kappa coefficient of 0.9. Metadata will be coded into different categories including outcomes and impacts using NVivo. Results of both quantitative and qualitative data analysis will be summarized in a searchable online database. Themes will be synthesized and explored in a narrative review and using simple logic models demonstrating theories of change for eligible case studies.

**Supplementary Information:**

The online version contains supplementary material available at 10.1186/s13750-022-00286-8.

## Background

Bamboo, a versatile commodity plant growing abundantly in many tropical, subtropical, and temperate climates, has been identified throughout sustainable development discourse as a potential source of climate-smart income generation for communities in bamboo-producing countries [1–3]. Due to this potential, numerous interventions have been implemented that aim to develop sustainable livelihoods with many environmental co-benefits through bamboo industry development. Entities that have implemented these strategies include the governments of several countries worldwide and international organizations like the International Fund for Agricultural Development (IFAD) and the International Bamboo and Rattan Organization (INBAR) [4].

These interventions can take many forms and use different strategies to promote the bamboo industry [5]. Examples of these strategies include training programs in bamboo plantation management, harvesting or product processing; policy changes to encourage the development of bamboo markets; raw material provision, including planting to meet joint environmental and social objectives; and direct funding for communities to invest in equipment and marketing. Still more projects aim to develop the bamboo industry indirectly through field trials into best practices for management and harvesting, research into the ecological benefits of bamboo plantations, and market research. Interventions may take place in collaboration with a range of stakeholders including universities, community groups, industry partners, and non-governmental organizations. Bamboo is a versatile material with many applications, so these types of projects range from construction, furniture, arts and handicrafts, food, cosmetics, or other industries. Specific target outcomes and impacts include a socio-economic component, including income increases, food security, and gender equality. These are usually in tandem with desired environmental benefits, such as increase in biodiversity, land restoration, watershed regulation, carbon sequestration, climate resilience, reducing reliance on timber resources, and promoting environmentally sustainable consumption.

**Fig. 1:**
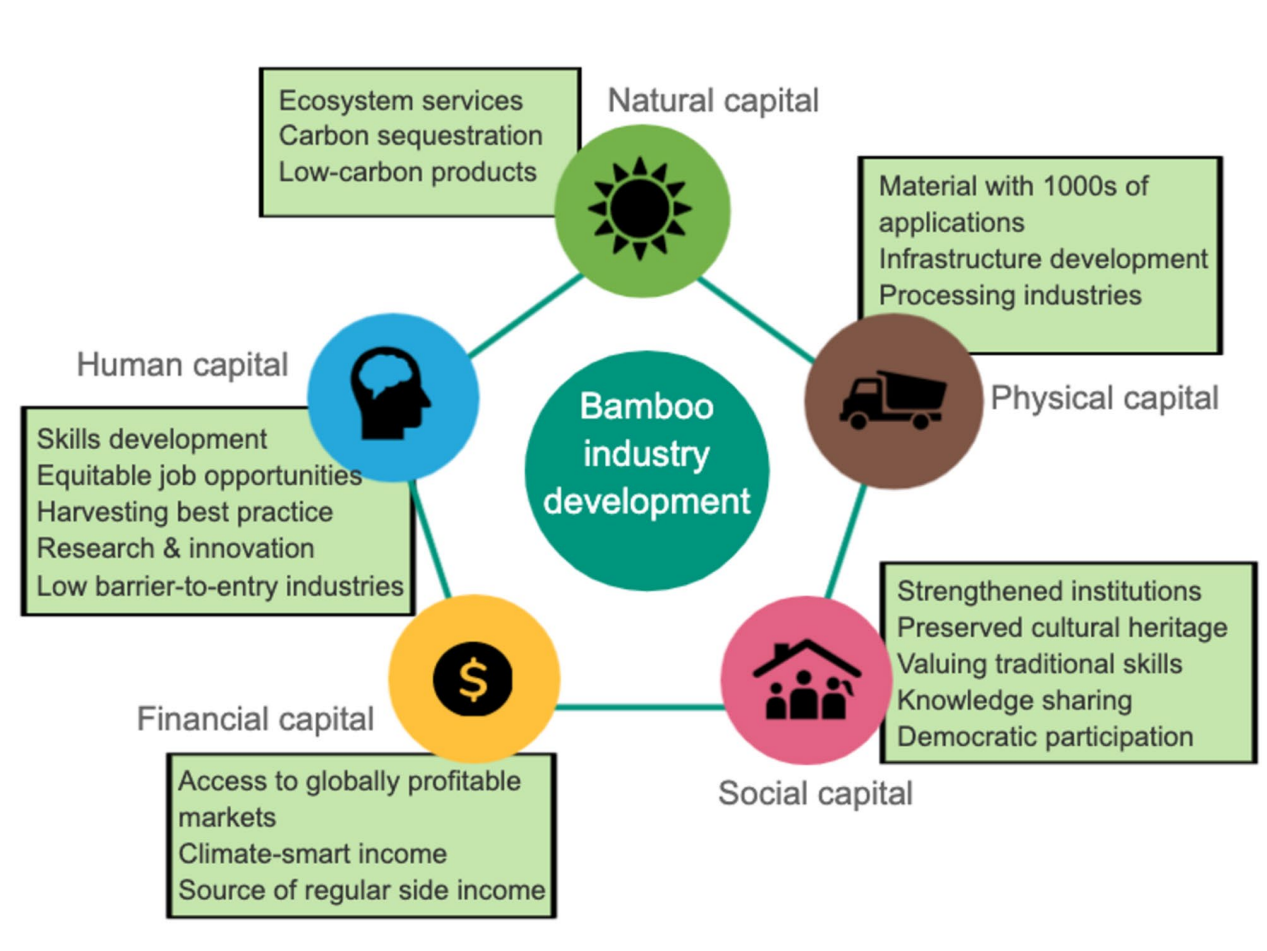
Bamboo industry development as an instrument for sustainable livelihood development. Adapted from Figs. 1, [25]

The rationale behind these initiatives is multifold and considers development of sustainable livelihoods through the accumulation of natural, physical, social, financial, and human capital (Fig. 1) [6]. Figure 1 contextualizes bamboo as a prospective nature-based solution to many social and environmental challenges.

Due to its fast growth, even on poor or degraded soils, bamboo plantations may sequester more carbon than other plants in a similar environment [7, 8]. Bamboo industry development is considered by many a climate resilient and inherently sustainable source of income, with many potential ecological co-benefits such as land restoration, watershed regulation and reduction in soil erosion [9, 10]. Bamboo grows well in some of the poorest areas of the world, and communities living in bamboo-producing areas may be some of the most vulnerable to climate change [11, 12]. Unlike trees, bamboo culms can be harvested many times on a shorter rotation cycle, providing faster income for farmers than equivalent tree plantations [13]. In just 4–7 years, many giant bamboo species can be ready for use in structural applications such as housing, whereas wood species used in the timber trade are generally harvested after several decades [14]. Also, unlike trees, bamboo is self-propagating, meaning that it can be harvested without killing the plant, encouraging regrowth, continually sequestering carbon and creating a regenerative income source for farmers over time [15]. Harvesting bamboo is therefore promoted as a strategy to reduce deforestation in areas with abundant bamboo resources and scarce timber availability. Knowledge of traditional crafts, processing and harvesting methods represents a valuable cultural heritage in bamboo-producing areas [16–19]. Many processing industries for bamboo, including hand weaving and the manufacture of household products, require little investment and can build on technical knowledge that already exists in rural communities, so they may provide a source of supplementary income for vulnerable communities [20, 21]. Artisanal industries such as the manufacture of bamboo charcoal or incense sticks, can often be carried out in the home [22, 23]. These industries may particularly benefit women, who disproportionately take on domestic tasks such as housework, cooking and child-rearing and face barriers to entering non-domestic industries [24].

Bamboo industry development is often framed as a vehicle for meeting many of the United Nations Sustainable Development Goals (SDGs), including but not limited to gender equality (SDG 5), economic growth (SDG 8), sustainable construction (SDG 11), climate change (SDG 13), and sustainable consumption (SDG 12). Some projects also consider important underlying social components such as participation, equity, and inclusion.

While much of the current literature is centered around the potential of bamboo to contribute to sustainable development and the SDGs in different capacities, some studies offer a more critical perspective that highlight the potential negative effects. Various unintended environmental impacts of bamboo plantations have been identified. These include the loss of biodiversity associated with the monocrop model employed in industrial bamboo plantations, and the invasive nature and rapid spreading of some bamboo species, particularly those that are not adequately managed in non-native ecosystems [26]. Other environmental critiques have focused more specifically on the loss of avian and soil diversity and an increase in landslides associated with large industrial bamboo plantations in China [27]. Some life cycle assessments have brought into question the commonly held assumption that bamboo products are more sustainable than alternatives, with particular focus on the impacts of manufacturing processes associated with bamboo fabric and paper on human health (28–29).

Few academic studies have specifically focused on the limitations and negative socio-economic impacts of bamboo industry development. However, potential barriers to success include lack of market access, distance to markets, lack of skills and technical resources, and lack of raw material. Other possible pitfalls of these interventions that are not unique to bamboo include the challenges of inter-institutional funding agreements, political corruption, and concerns over equitable pay and working conditions for bamboo producers/harvesters. Some research into the impact of the commercialization of non-timber forest products (NTFP), which include bamboo, has highlighted that case studies of NTFP commercialization showing substantial benefit to local people are rare in practice despite considerable investment and interest in the field [30].

Much of the current published peer-reviewed literature on bamboo for livelihood development discusses the theoretical potential of the sector to meet social, economic, and environmental objectives [31–35]. Other related literature on outcomes and impacts is currently in the form of individual case studies, or project reports written by organizations that have implemented or funded these projects. Notably, INBAR has published many organizational reports on the benefits of bamboo industry development [36–39]. These sources report outcomes of the interventions in a myriad of ways, and there is no universally established metric for measuring impact. Many of these reports have a significant incentive to emphasize positive outcomes over negative or neutral ones, since further funding or the continuation of the project may be contingent on positive outcomes [40]. These texts nevertheless represent a body of evidence that has so far not been comprehensively studied.

There have been no systematic attempts to summarize evidence for the effectiveness of these initiatives. Establishing this evidence base is an essential step towards identifying where funding, research or further efforts in this sphere would be best concentrated. The proposed systematic map takes a broad approach covering all industries and strategies, focusing on social, environmental, and economic outcomes. Data analysis focusing on only one sector or strategy will be considered for further research.

### Stakeholder Engagement

The proposed systematic map will be carried out by researchers from the University of British Columbia, with input from collaborators at the University of Alberta, the University of Western Ontario and Royal Roads University. Experts from the World Bamboo Organization, the International Bamboo and Rattan Organization, the International Centre for Bamboo and Rattan, and individuals from the worlds of international development, government and industry may be consulted during this process by email to add sources that may have been missed from the review and to check the accuracy of documents during the writeup process.

## Objective of the review

The objective of this proposed systematic map is to identify and evaluate the evidence base on the social, environmental, and economic impacts and outcomes from interventions that aim to promote sustainable livelihoods through bamboo industry development. Key elements of the study focus are described in Table 1 below.

The systematic map aims to answer the following central research question: What is the evidence on the social, environmental and economic impact of interventions that facilitate bamboo industry development for sustainable livelihoods?

**Table 1:** PIO elements for formulating research question

PIO element	
**Population**	Global projects concluded since 1990 that promote bamboo industry development
**Intervention**	Projects with a primary aim of livelihood development or other similar social objectives.
**Outcome**	Social, environmental, and economic outcomes

Secondary research questions are:

- What are the critical success factors for these projects and how is this success measured?
- What are strategies that have not been successful and why?
- What are the theories of change for these projects?
- What are the gaps or poorly understood aspects in this literature for further research?

## Methods

ROSES forms and reporting standards were followed and a ROSES form for systematic map protocols is attached as Additional File 1 [41]. The protocol also adheres to Collaboration for Environmental Evidence (CEE) guidelines for authors [42].

### Searching for articles

Searches for relevant articles will be carried out via three main avenues with different search terms and searching strategies associated with each method.

#### Searching languages

English will be the searching language of this review. Documents in other languages will be saved and stored for future review.

#### Databases to be searched

We will such for peer-reviewed literature using the Web of Science, PubAg and Cab Direct bibliographic databases using institutional logins from the institutions of the reviewers.

#### **Grey literature**

Grey literature sources for this map include program evaluations and project documents found on the websites of international organizations that work in the target area. A list of these organizations for inclusion can be found using the Yearbook of International Organizations [43]. Table 2 shows a list of such organizations from a preliminary search using keywords relating to bamboo, agriculture, forestry, livelihoods, and the environment. These organizational websites, and any others that are deemed appropriate from related searches, will be used to retrieve sources from the online resource library, project reports or equivalent section of the website.

**Table 2:** International organizations to be included in the search of grey literature. Other organizations may be added throughout the search

Organization	Website
International Bamboo and Rattan Organization (INBAR)	www.inbar.int
International Fund for Agricultural Development	www.ifad.org
World Bank	www.worldbank.org
Spanish Agency for International Development Cooperation (AECID)	www.aecid.es
United Nations Development Programme (UNDP)	www.undp.org
United Nations Environment Programme	www.unep.org
World Food Programme	www.wfp.org
Deutsche Gesellschaft für Internationale Zusammenarbeit (GIZ)	www.giz.de
International Union for the Conservation of Nature (IUCN)	www.iucn.org
The Nature Conservancy	www.nature.org
European Environment Agency	www.eea.europa.eu
The Rainforest Alliance	www.rainforest-alliance.org
Global Landscapes Forum	www.globallandscapesforum.org
Worldwide Fund for Nature (WWF)	www.worldwildlife.org
United Nations Food and Agriculture Organization (FAO)	www.fao.org
World Trade Organization (WTO)	www.wto.org
International Union of Forest Research Organizations (IUFRO)	www.iufro.org
United Nations Educational, Scientific and Cultural Organization (UNESCO)	www.unesco.org
United Nations Conference on Trade and Development (UNCTAD)	www.unctad.org
UN Convention on Biodiversity	www.cbd.int

#### Other sources

Google Scholar will be used as a supplementary source to retrieve both peer-reviewed and grey literature [44]. The software tool Publish or perish) will be used to retrieve sources from Google Scholar. Finally, experts from important stakeholder groups will be consulted to add documents that the original search may have missed.

#### Search terms

Each type of database will require a slightly different combination of search terms due to the specific content requirements of the database. Search strings were devised by defining PIO question elements, resulting in the research question detailed in “Objective of the Review” above. To ensure that results containing the Latin names of bamboo species could be found, rather than solely those containing the word “bamboo”, the Latin names of bamboos were added to “Population” terms. The list of Latin names used were taken from a list of the most common priority bamboo species [45], since most bamboo species are not commonly used in livelihood development. All search results should contain the word “bamboo” or “bamboos”, or an equivalent Latin name for a bamboo species, in the title, abstract, or keywords. The results should also contain at least one of the terms from the “Intervention term,” “Outcome term” and “Change term” rows in Table 3. The columns contain both general and specific terms to search for known and hitherto unknown interventions and outcomes. These groups of terms were included in parentheses with the operator OR between them and added to the search string with the operator AND between the groups.

**Table 3:** Search strings as formatted for the Web of Science database

*1. Population terms*	Bamboo* OR Bambusa OR Dendrocalamus OR Gigantochloa OR Guadua OR Melocanna OR Ochlandra OR Phyllostachys OR Thyrsostachys OR Schizostachyum OR Arundinia OR Lingnania OR Oxytenthera OR Chusquea
	AND
*2. Intervention Terms*	socioeconomic OR socio-economic OR rural OR empower* OR communit* OR econom* OR “value chain*” OR “cultural heritage” OR “traditional knowledge” OR industr* OR livelihood* or financ* OR poverty OR income* OR inclus*
	AND
*3. Outcome terms*	Climat* OR outcome* OR result* OR impact* OR social* OR “food security” OR gender* OR environment* OR contribut* OR ecolog* OR evaluat* OR benefit* OR effect* OR “global warming” OR “land restoration” OR soil* OR water* OR air OR capacit* OR particip*
	AND
*4. Change terms*	Change* OR relation* OR develop* OR affect* OR project* OR program* OR interven* OR initiative* OR implement*

One of the databases, PubAg, does not allow for publication year to be included in the search string. Results will be filtered by publication year after the searching step. PubAg also does not support wildcards or truncations. Due to capacity limitations when using the Google search enginer, the simplified search keyword combinations shown in Table 4 will be used in Google [46]. A further search for pdf documents published on organizational websites will take place using the search terms found in Table 4 in the Google search engine to find any pdf files hosted on organizational websites containing the word “bamboo”.

**Table 4:** Summary of specific search strings used in different databases

Database	Search string	Additional filters/details
**Web of Science**	(((TS=(bamboo* OR Bambusa OR Dendrocalamus OR Gigantochloa OR Guadua OR Melocanna OR Ochlandra OR Phyllostachys OR Thyrsostachys OR Schizostachyum OR Arundinia OR Lingnania OR Oxytenthera OR Chusquea)) AND TS=(socioeconomic OR socio-economic OR rural OR empower* OR communit* OR econom* OR “value chain*” OR “cultural heritage” OR “traditional knowledge” OR industr* OR livelihood* or financ* OR poverty OR income* OR inclus*)) AND TS=(Change* OR relation* OR develop* OR affect* OR project* OR program* OR interven* OR initiative* OR implement*)) AND TS=(Climat* OR outcome* OR result* OR impact* OR social* OR “food security” OR gender* OR environment* OR contribut* OR ecolog* OR evaluat* OR benefit* OR effect* OR “global warming” OR “land restoration” OR soil* OR water* OR air OR capacit* OR particip*)	Publication Date = 1990-01-01 to 2021-12-31
**CAB Direct**	(((bamboo*) OR (bambusa) OR (dendrocalamus) OR (gigantochloa) OR (guadua) or (melocanna) or (ochlandra) OR (phyllostachys) OR (thyrsostachys) OR (schizostachyum) OR (arundinia) OR (lingnania) OR (oxytenthera) OR (chusquea)) AND ((inclus*) OR (socioeconomic) OR (“value chain”) OR (poverty) OR (livelihood*) OR (industri*) OR (income*) OR (social*) OR (particip*) OR (“cultural heritage”) OR (“traditional knowledge”) OR (econom*) OR (empower*) OR (socio-economic) OR (rural) OR (communit*) ) AND ((develop*) OR (change*) OR (affect*) OR (relationship*) OR (interven*) OR (project*) OR (program*) OR (initiative*) OR (implement*)) AND ((evaluat*) OR (social*) OR (outcome*) OR (result*) OR (effect*) OR (impact*) OR (climat*) OR (environment*) OR (ecolog*) OR (benefit*) OR (contribut*) OR (gender*) OR (“food security”) OR (“global warming”) OR (“land restoration”) OR (soil*) OR (water*) OR (air) OR (capacit*) OR (particip*)) AND yr:[1990 TO 2021])	Publication Year = 1990 to 2021
**PubAg**	(bamboo OR Bambusa OR Dendrocalamus OR Gigantochloa OR Guadua OR Melocanna OR Ochlandra OR Phyllostachys OR Thyrsostachys OR Schizostachyum OR Arundinia OR Lingnania OR Oxytenthera OR Chusquea) AND (inclusion OR inclusive OR financial OR socioeconomic OR “value chain” OR poverty OR livelihood OR industry OR industrial OR “cultural heritage” OR “traditional knowledge” OR economy OR economic OR empower OR socio-economic OR rural OR community OR income) AND (development OR change OR affect OR intervention OR project OR program OR initiative OR implement) AND (evaluation OR social OR outcome OR result OR effect OR impact OR climate OR environment OR ecological OR ecology OR benefit OR contribute OR contribution OR gender OR “food security” OR “global warming” OR “land restoration” OR soil OR water OR air OR capacity OR participation OR participate)	Publication Year = 1990 to 2021
**Organizational websites excluding INBAR**	bamboo	
**INBAR website**	value chain	
	Inclusion	
	inclusive	
	poverty	
	livelihood	
	industry development	
	income	
	social impact	
	cultural heritage	
	project	
	program evaluation	
	traditional knowledge	
	economy	
	economic	
	empowerment	
	rural	
	socioeconomic	
	socio-economic	
**Google to search in organizational websites**	site:example.com filetype:pdf “bamboo”	“example.com”= organizational website
**Google (first ten pages of search results only)**	(bamboo*) AND (inclus* OR financial OR socioeconomic OR “value chain” OR poverty OR livelihood* OR indust* OR econom* OR empower* OR socio-economic OR rural OR communit* OR income*) AND (develop* OR change* OR affect* OR interven* OR project* OR program* OR initiative* OR implement*) AND (evaluat* OR social OR particip* OR outcome* OR result* OR effect* OR impact* OR climate* OR environment* OR benefit* OR contribut* OR gender* OR soil* OR water*)	Google search results are limited to 32 words
**Google Scholar**	bamboo (poverty | socioeconomic | economic | industry | finance | income | benefit | capacity | livelihood | intervention | evaluation | impact | outcome | change | develop | project | program | effect | community | social)	

Search terms for organizational websites differ from those used in the bibliographic databases. Since publications authored by specific organizations are more limited than those found in larger databases, the word “bamboo” suffices as a search term. An exception is the publications library of the International Bamboo and Rattan Organization, which contains many publications on bamboo. For this database, keywords from Column 2 in Table 3 will be used. Since this database does not support searches using Boolean Operators, the keywords will be used individually.

The academic web-based search engine Google Scholar will be used as an additional sources to retrieve both peer-reviewed and grey literature. Due to a character limit of 256 characters including spaces for searches, a shortened search term will be used for searching in Google Scholar. Google Scholar does not permit truncated searches, nesting using parentheses with more than one level, or wildcards, but it does include synonyms of search terms. A unique search string for use in Google Scholar is shown in Table 4.

#### Comprehensiveness testing

Search strings were tested for comprehensiveness using a list of 14 benchmark articles. These benchmarks articles are known peer-reviewed case studies that fit the search criteria. Through an iterative process, search strings were improved by adding and removing search terms, altering Boolean Operators and adding wildcards and truncation where necessary and possible to retrieve all selected benchmark articles. Across all three databases, all 14 benchmark articles were found using the chosen final search string. Since all the articles were retrieved, this search string was judged to be sufficiently comprehensive. Specific final search strings used are provided in Additional File 2 and in Table 4. Final search strings shown in Table 4 after comprehensiveness and sensitivity testing are a combination of the terms in Table 3 and Boolean Operators and special characters for documents published between 1 and 1990 and 31 December 2021.

## Article screening and study eligibility criteria

### Screening process

We will use a two-step screening process: (1) at the title-abstract level and (2) at the full article level.

#### Title-abstract consistency checking and screening

Reviewers will screen titles and abstracts and train themselves on batches of 30 articles to ensure consistency across reviewers. Using the Kappa statistical test for interrater reliability, if the Kappa coefficient between the two reviewers does not reach 0.9, then discussion between the two reviewers and possible tweaking of the eligibility criteria will ensue. Then, the reviewers will test consistency again until a Kappa coefficient of 0.9 has been reached, at which point the screening process can begin in earnest [47]. During the screening process, all sources will be screened for eligibility by two reviewers at the title-abstract level at the same time.

During the screening process, disagreements between reviewers on the screening process can be resolved through discussion between the two reviewers to decide whether the source in question should be included or excluded. In cases in which no agreement can be reached, a third reviewer will be brought for a tie-breaker vote.

#### Full-text screening and consistency checking

At the full text level, articles will be skimmed for eligibility according to the study eligibility requirements listed in the “eligibility criteria” section of this protocol”. Reviewers will first test consistency again using the Kappa interrater reliability test and batches of 30 articles. When a suitable Kappa coefficient (> 0.9) has been reached, then reviewers will review articles individually. Disagreements can be resolved by discussion between the two reviewers.

#### Grey literature screening

The screening process for grey literature sources will differ from that of the bibliographic databases. Only the title and a preview of the file will be visible upon first viewing to the reviewers, The first screening stage will therefore be at the title level only [48]. The second screening stage will involve skimming the entire article or document to ascertain eligibility. If the document is over five pages long, then a whole-document search for the word “bamboo” will be used to find the relevant section.

#### Google and other sources

Due to the large number of results found using the Google search engine, this search will be limited to the first ten pages of Google search results to find the most relevant results whilst still maintaining reviewer feasibility [49]. Reviewers will skim the title and preview of the search result before deciding whether to review the full result. The double screening process described above for the title-abstract screening stage will also apply to Google search results.

#### Reviewer bias

No reviewers that have co-authored articles to be considered as sources in the review will participate in the reviewing process, so there is no risk of reviewer bias.

### Eligibility criteria

A list of excluded full texts and reason for exclusion will be provided with the publication of the systematic map. The following criteria will be used to ascertain whether texts are to be included or excluded from the review.

#### Eligible populations or subjects

To be included, the source must describe efforts to develop the bamboo industry, through training, funding, capacity building, institutional support, or any other similar methods. Beneficiaries from any country or region and at any scale, including regional, national, and international, will be included. Any beneficiary group, including youth, women, farmers, and professionals, will be included, since the aim is to review evidence on interventions that promote or develop the bamboo industry, without focusing on any group.

#### Eligible intervention(s)

Interventions with a primary aim of bamboo industry development or industry promotion will be included. Interventions that concluded between 1990 and 2021 will be included to encompass the period over which interest in bamboo as a tool for livelihood development in the international sphere has grown and developed. Interventions in any country/region implemented by stakeholders or partners from various groups, including government, international agencies, non-governmental organizations, community groups, and other similar organizations will be included.

#### Eligible comparator(s)

In qualitative research the comparator is often implicit, so any comparator, including none, will be considered as eligible.

#### Eligible outcomes

Interventions with livelihood development or other related social outcomes as an aim will be included. Interventions with purely environmental objectives, such as conservation, land restoration or carbon sequestration, without any human or social element, are to be excluded, as these do not encompass industry development interventions, but interventions with both social and environmental objectives are to be included. Interventions that report environmental, social, or economic outcomes or impacts using any indicators are to be included. Projects that report no outcomes but describe an eligible intervention will be included and reported as such. This project uses definitions of “outcomes” and “impacts” first defined by the Center for International Forestry Research (CIFOR), which differentiate clearly between the two terms as referring to different types of change (Table 5) [50].

**Table 5:** Categories of data to be collected in the coding stage

Category	Definition	Example		Subcategories	
1. Country	Country or countries where the intervention was carried out	China		1a. Asia 1b. Africa 1c. South America 1d. Europe 1e. Central America 1 f. North America 1 g. Australia	
2. Intervention begins year	Year in which the intervention began	1996		2a. 1990–1994 2b. 1995–1999 2c. 2000–2004 2d. 2005–2009 2e. 2010–2014 2 f. 2015–2020 2 g. 2021–2022	
3. Intervention ends year	Year in which the intervention ended	2000		3a. 1990–1994 3b. 1995–1999 3c. 2000–2004 3d. 2005–2009 3e. 2010–2014 3 f. 2015–2020 3 g. 2021–2022	
4. Implementing body	Name of the organization or organizations that planned and implemented the relevant project, and type of organization	International Union for the Conservation of Nature		4a. Government 4b. Non-governmental organization 4c. Community Organization 4d. International Organization	
5. Beneficiaries	Group of people or communities that the intervention aimed to benefit	Young people aged 18–25		5a. Farmers 5b. Rural community 5c. Urban community 5d. Youth 5e. Women	
6. Industry	Specific bamboo product or activity	Furniture		6a. Plantations 6b. Bamboo shoots 6c. Construction 6d. Furniture 6e. Handicrafts	
7. Objective	Stated goal of the project	To increase average incomes in a particular area through training on planting bamboo		n/a	
8. Baseline Study	Existence or otherwise of a study into conditions before the intervention took place	Yes; surveys measuring current state of ecosystem and participant income sources, health and education level carried out		8a. Yes 8b. No	
9. Input	Resources	Government grant		9a. Government funding 9b. University funding 9c. Private sector funding 9d. Other	
10. Participatory processes	Strategy including elements of democratic participation	Participatory meetings with project beneficiaries held before, during and after project implementation		10a. Yes 10b. No	
11. Interventions	The activities which make up the project implementation	Training sessions held		11a. Product training 11b. Direct funding 11c. Credit 11d. Policy change 11e. Lobbying 11 f. Strengthening institutions 11 g. Plantation management 11 h. Research	
12. Outputs	The direct, measurable results of the intervention	hectares of bamboo planted		n/a (freeform field, no categories)	
13. Outcomes	“[A] change in knowledge, attitudes and/or skills, manifest as a change in behavior that results in whole or in part from the… [intervention]… and its outputs” (52).	Increase in an individual income as a direct result of the project		13a. Participation and engagement 13b. Individual of community wellbeing 13c. Financial security 13d. Food security 13e. Health 13 f. Gender equality 13 g. Sustainable consumption patterns 13 h. Social equity	
14. Impact	“[A] change in flow or a change in state resulting in whole or in part from a chain of events to which… [the intervention] … has contributed” (52). Impacts are usually measured using change in a specific parameter, like income, air quality or number of jobs.	Decrease in income inequality; increase in soil and air quality		14a. Economic 14b. Social 14c. Environmental 14d. Physical 14e. Cultural 14 f. Institutional	
15. Critical success factor	A precondition of the project succeeding in its stated goals	Engaged and cooperative government stakeholders		n/a (freeform field, no categories)	
16. Evaluation indicator	A way to measure outcomes and progress	Number of people that attended an event		16a. Financial indicators 16b. Human indicators 16c. Environmental indicators 16d. Social indicators 16 f. Cultural indicators 16 g. Physical indicators	
17. Evaluation methodology and details	How outcomes and progress were measured and how many participants were involved in the evaluation process	Theory of change; cost-benefit analysis		n/a (freeform field, no categories)	

#### Eligible types of study design

Since there is no universally agreed upon program evaluation methodology for this type of intervention, any study design will be considered for inclusion.

### Study validity assessment

As per systematic map methodology [51], study validity assessment will not be conducted in detail at this stage. However, information about the study corresponding to the categories shown in Table 5 below will be stored and may be used to develop a set of comprehensive criteria to allow the validity of this type of study to be assessed in the future. This includes information about the presence of a baseline study, information about the specific evaluation methods and indicators used, and details about the length of evaluation period and number of participants included in evaluations.

### Data coding strategy

Once the final list of relevant sources has been obtained, then two reviewers will independently code one source and informally compare coding to highlight and resolve any large discrepancies or misunderstandings in coding, and if needed adjust the metadata coding strategy and coding frame. Sources will be coded using the Nvivo software package. Metadata corresponding to one of the preliminary categories (“parent nodes” in Nvivo) and subcategories (“codes” in Nvivo) in Table 5 will be collected. After the first round of consistency checking, a second round of 10 sources will be double coded independently with a goal of reaching agreement on 80% of the number and content of codes, the two reviewers will be compared [51]. If this goal is not reached, then another round of discussion, adjustment of coding strategy and frame, and possible adjudication from a third reviewer will be undertaken. Another role of 10 sources will then be double coded independently, until this goal is reached.

As analysis continues after the consistency checking exercise, remaining sources will be coded independently. Additional themes of interest may be noted and coded using Nvivo software in an iterative process of thematic synthesis [52]. New codes will be added to a shared document visible to both reviewers to avoid superfluous codes being created, and these codes will also be accessible to both reviewers via the software. Since several sources may refer to the same intervention, then each project or intervention will be recorded as a separate “Case” in Nvivo. Information about each project will be recorded in an Excel spreadsheet with one row representing one project, and one column representing each category from Column 1 in Table 5. Table 5 shows categories to be coded and subcategories to be included along with details and examples for clarity.

Missing, incomplete or unclear information will be noted, but no attempt will be made at this stage to obtain more information from authors. This exercise will be considered for further research.

### Study mapping and presentation

The resulting database from the coding phase will be made available as part of the systematic map. The most common theme, critical success factors and outcomes will be listed. Then, simple quantitative analysis such as frequency analysis will be used to describe and quantify trends in the data. Qualitative analysis in the form of a narrative review will further describe and compare case studies, giving detailed examples of the most common themes. Notable gaps and areas of research paucity will be described. In addition, for selected representative and data-rich projects, a theory of change in the form of a simple logic model will be presented and discussed showing critical success factors and logical pathway from input to output [53, 54]. Elements to be included in these schematics include input, intervention, output, outcomes and impact, as defined in Table 5. The purpose of this logic model method is to provide a theoretical basis for comparison and understanding of case studies, while accounting for the many differences in evaluation methods and reporting standards adhered to in the texts analyzed for the proposed study.

## Electronic supplementary material

Below is the link to the electronic supplementary material.

Supporting information 1: Search string

Supporting information 2: ROSES form

## Data Availability

Not applicable.
